# Supplementary material for: Microsyntenic Clusters Reveal Conservation of lncRNAs in Chordates Despite Absence of Sequence Conservation
Source: Biology (Basel). 2019 Aug 24;8(3):61. doi: 10.3390/biology8030061 (PMC6784235; doi:10.3390/biology8030061)
Supplement: Supplementary file 1 [file biology-08-00061-s001.zip › biology-568094-SI/Supplementary tables.pdf]

Table S1. Putatively conserved lincRNAs between *Homo sapiens* and *Branchiostoma lanceolatum.* Analysis of the genes surrounding the lincRNA focusing on three genes (upstream2, upstream1 and lincRNA). Some lincRNAs can be adscribed to more than one hypothetically conserved microsyntenic clusters.

**Orthologous lincRNAs[1]**	**State of the cluster in human[2]**	**Human orthologous lincRNA[3]**
BL02382|Sc0000007|7|+|linc	Synteny conserved in the coding genes	AC005258.2
BL57655|Sc0000007|114|+|linc	*Conserved microsynteny	LOC100505666
BL58800|Sc0000008|5|-|linc	One gene with the strand inverted	LINC01124<br>AC007405.1
BL62558|Sc0000008|145|-|linc	*Conserved microsynteny	TCONS_l2_00003921
BL42493|Sc0000014|4|+|linc	*Conserved microsynteny	TCONS_00016098
BL39635|Sc0000017|32|-|linc	Two genes with the strand inverted	LOC101448202
BL76639|Sc0000022|35|+|linc	One gene with the strand inverted	SULT1C2P1
BL81207|Sc0000033|4|-|linc	Synteny conserved in the coding genes	TCONS_00019766
BL63533|Sc0000034|21|+|linc	*Conserved microsynteny but extra gene in human	LINC01877
BL79865|Sc0000034|22|-|linc	*Conserved microsynteny but extra gene in human	TCONS_00002678
BL42364|Sc0000034|23|-|linc	*Conserved microsynteny but extra gene in human	AC097717.1
BL34463|Sc0000054|53|-|linc	Correct order but strands inverted	AL355338.2
BL56140|Sc0000081|24|-|linc	Synteny conserved in the coding genes	AC012309.2
BL88002|Sc0000083|10|-|linc	*Conserved microsynteny	AC007298.2
BL70460|Sc0000095|4|+|linc	Two genes with the strand inverted	TCONS_00018032
BL35238|Sc0000141|19|-|linc	One gene with the strand inverted	TCONS_00025555
BL43288|Sc0000247|8|+|linc	Two genes with the strand inverted	NM_001302493.1
BL83080|Sc0000266|1|-|linc	*Conserved microsynteny	AC115284.2
BL66360|Sc0000273|1|-|linc	*Conserved microsynteny	AC012354.3
BL89644|Sc0000273|2|-|linc	*Conserved microsynteny	LINC01833
BL26716|Sc0000273|3|-|linc	*Conserved microsynteny	SIX3-AS1
BL68659|Sc0000384|9|-|linc	*Conserved microsynteny	TCONS_00011917

[1]GenID, Scaffold, virtual coordinates and strand separated by "|". [2]Description of the synteny of the cluster status in human. [3]ID of the putative human orthologous lincRNA. * indicates a perfect match in strand and order of the three core genes.

Table S2. Putatively conserved lincRNAs between *Homo sapiens* and *Branchiostoma lanceolatum.* Analysis of the genes surrounding the lincRNA focusing on three genes (lincRNA, downstream1 and downstream2). Some lincRNAs can be adscribed to more than one hypothetically conserved microsyntenic clusters.

Orthologous lincRNAs[1]	State of the cluster in human[2]	Human orthologous lincRNA[3]
BL48403|Sc0000000|10|-|linc	Synteny conserved in the coding genes	AC004160.1
BL91553|Sc0000000|32|+|linc	*Conserved microsynteny	HOXB-AS1
BL83549|Sc0000000|42|-|linc	Synteny conserved in the coding genes	TCONS_00003443<br>HOXB-AS4
BL49148|Sc0000004|180|+|linc	Two genes with the strand inverted	TCONS_00016695
BL88007|Sc0000005|228|+|linc	*Conserved microsynteny	AC009159.2
BL88764|Sc0000006|154|+|linc	Correct order but strands inverted	TCONS_l2_00025400
BL21777|Sc0000012|28|+|linc	*Conserved microsynteny	KCNJ2-AS1
BL43485|Sc0000012|77|+|linc	*Conserved microsynteny but extra gene in human	TCONS_00025331
BL38852|Sc0000015|95|+|linc	Two genes with the strand inverted	TCONS_00003896
BL23696|Sc0000017|69|+|linc	One gene with the strand inverted	AC100825.1
BL78928|Sc0000022|40|-|linc	One gene with the strand inverted	Z98257.1
BL61801|Sc0000024|6|+|linc	Two genes with the strand inverted	AC092364.1
BL41653|Sc0000050|7|+|linc	Two genes with the strand inverted	TCONS_00014852
BL64814|Sc0000066|36|-|linc	Synteny conserved in the coding genes	Z98880.1
BL70524|Sc0000080|28|+|linc	*Conserved microsynteny	AC114812.2
BL84269|Sc0000083|14|+|linc	*Conserved microsynteny	AC090001.1
BL66046|Sc0000083|15|-|linc	*Conserved microsynteny	LINC02410
BL62840|Sc0000091|33|+|linc	*Conserved microsynteny	AL158013.1
BL90962|Sc0000097|13|-|linc	Synteny conserved in the coding genes	AC012254.3
BL67055|Sc0000174|7|-|linc	Two genes with the strand inverted	AC006398.1
BL33122|Sc0000193|3|-|linc	Synteny conserved in the coding genes	LINC00210
BL49481|Sc0000194|3|-|linc	One gene with the strand inverted	AC092364.1
BL57370|Sc0000219|7|+|linc	*Conserved microsynteny	LINC01438
BL52347|Sc0000273|6|-|linc	Synteny conserved in the coding genes	TCONS_00022526
BL52353|Sc0000273|7|-|linc	*Conserved microsynteny	AC093702.1

[1]GenID, Scaffold, virtual coordinates and strand separated by "|". [2]Description of the synteny of the cluster status in human. [3]ID of the putative human orthologous lincRNA. * indicates a perfect match in strand and order of the three core genes.
